# Supplementary material for: Successful delivery of large-size CRISPR/Cas9 vectors in hard-to-transfect human cells using small plasmids
Source: Commun Biol. 2020 Jun 19;3:319. doi: 10.1038/s42003-020-1045-7 (PMC7305135; doi:10.1038/s42003-020-1045-7)
Supplement: Supplementary file 1 — Supplementary Information [file 42003_2020_1045_MOESM1_ESM.pdf]

Supplementary Figures

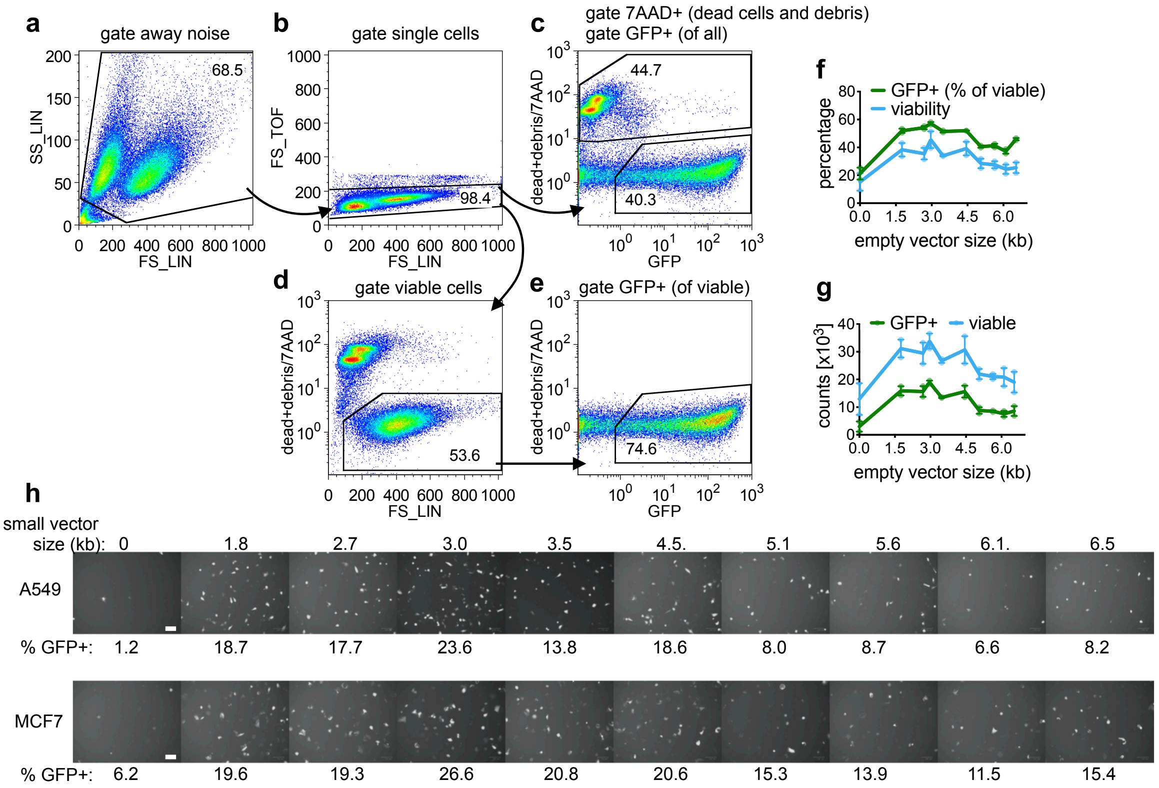

Supplementary Fig. 1: Co-transfection of a 3kb small vector results in the highest transfection efficiencies. **a-e**, Exemplified gating strategy for flow cytometry analysis of GFP (transfection efficiency) and 7AAD (dead cell marker) shown for A549 cells. The number of GFP+ cells are represented either as percentage of all cells (as in **c**) or as percentage of viable cells (as in **e**). For all experiments, the cell count within the GFP+ gate is provided in the supplementary figures and corresponds to the number of acquired events in 120 seconds at a steady flow rate within the indicated gate. The 7AAD gate will contain both recently dead cells as well as subcellular debris, which may distort the measurement of actual viability as one dead cell may break down into several debris. **f-g**, Line graphs illustrates (**f**) percentage and (**g**) number of GFP+ cells (green) and cell viability (blue) upon co-transfection of a large 15kb vector with small vectors of varying sizes (1.8-6.5kb) in A549 and MCF7 cells (n=4, mean +/- SEM). **h**, Microcopy images assaying GFP expression (white dots) for A549 (top) and MCF7 (bottom) electroporated with a 15kb CRISPR-GFP vector and a small vector of the size indicated above. Number below each microscopy image indicates percentage of viable GFP+7AAD- cells as determined by flow cytometry. Scale bar: 100 µm.

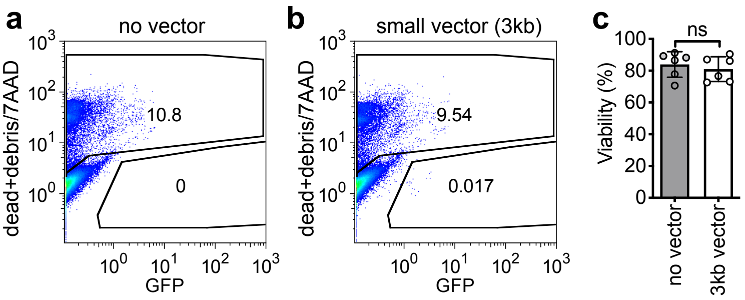

Supplementary Fig. 2: The small vector does not affect cell viability by itself. Flow cytometry plots (**a-b**) and bar graph (**c**) illustrate quantification of cell viability comparing electroporation with or without a small (3kb) vector (n=6, mean + SEM). Statistics: paired two-tailed t-test, ns: not significant.

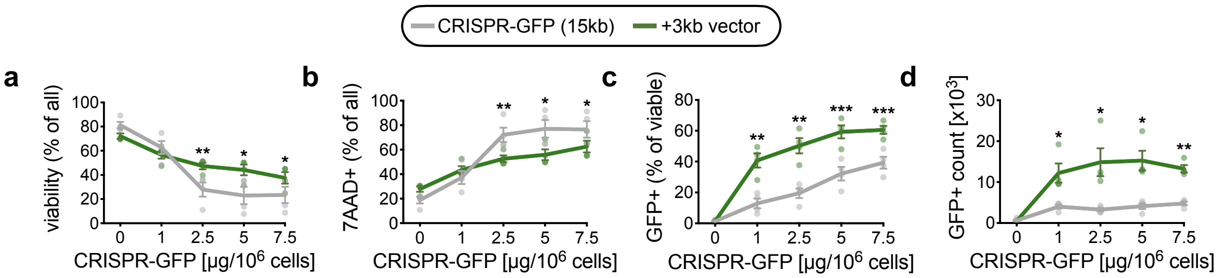

Supplementary Fig. 3: The small vector improves transfection efficiencies irrespective of the concentration of the large CRISPR-GFP vector. **a-d**, Line graphs demonstrate the percentage and number of viable, GFP+, and 7AAD+ (dead and debris) cells after co-transfection of varying amounts (0-7.5 µg) of a large CRISPR-GFP vector (15kb) without (grey, 0 µg) and with (green, 5 µg) a small vector (3kb) in A549 and MCF7 cells (n=4, mean +/- SEM). Two controls consisting of no vector and only the small vector represent the cell percentage or count when transfecting 0 µg of the large CRISPR-GFP vector (x-axes). Statistics: paired two-tailed t-test, *$p < 0.05$, **$p < 0.01$, ***$p < 0.001$.

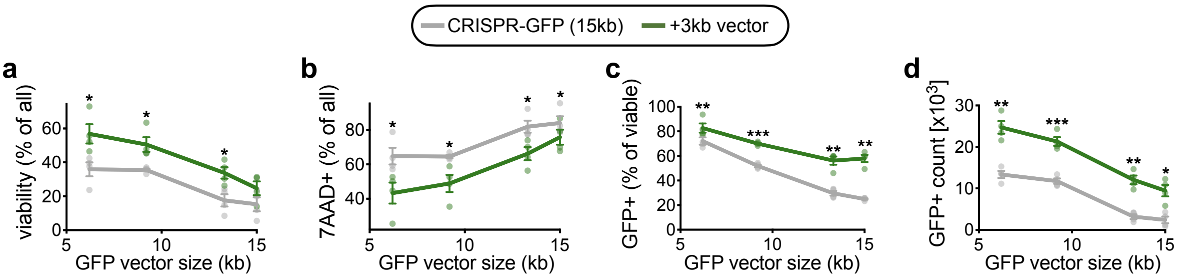

Supplementary Fig. 4: The small 3kb vector improves cell viability and transfection efficiency for varying sizes of large GFP vectors. **a-d**, Line graphs demonstrate the percentage and number of GFP+ and viable cells after co-transfection of large GFP vectors (6.5-15kb) without (grey) and with (green) a small vector (3kb) in A549 and MCF7 cells (n=4, mean +/- SEM). Statistics: paired two-tailed t-test, *$p < 0.05$, **$p < 0.01$, ***$p < 0.001$.

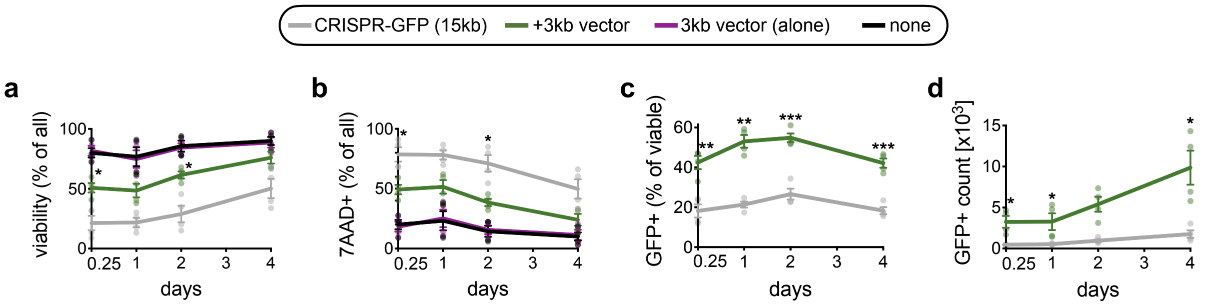

Supplementary Fig. 5: The small 3kb vector improves cell viability and transfection efficiency for several days. **a-d**, Line graphs demonstrate the percentage and number of viable, GFP+, and 7AAD+ (dead and debris) cells after co-transfection of a large CRISPR-GFP vector (15kb) without (grey) and with (green) a small vector (3kb) in A549 and MCF7 cells at time points from 6h (0.25d) until day four after transfection (n=4, mean +/- SEM). Cell transfection controls with neither large nor small vector (black) and only the small 3kb vector alone (purple) are shown in **a-b**. Statistics: paired two-tailed t-test comparing the large CRISPR-GFP vector with and without the small vector (green versus grey curves), *$p < 0.05$, **$p < 0.01$, ***$p < 0.001$.

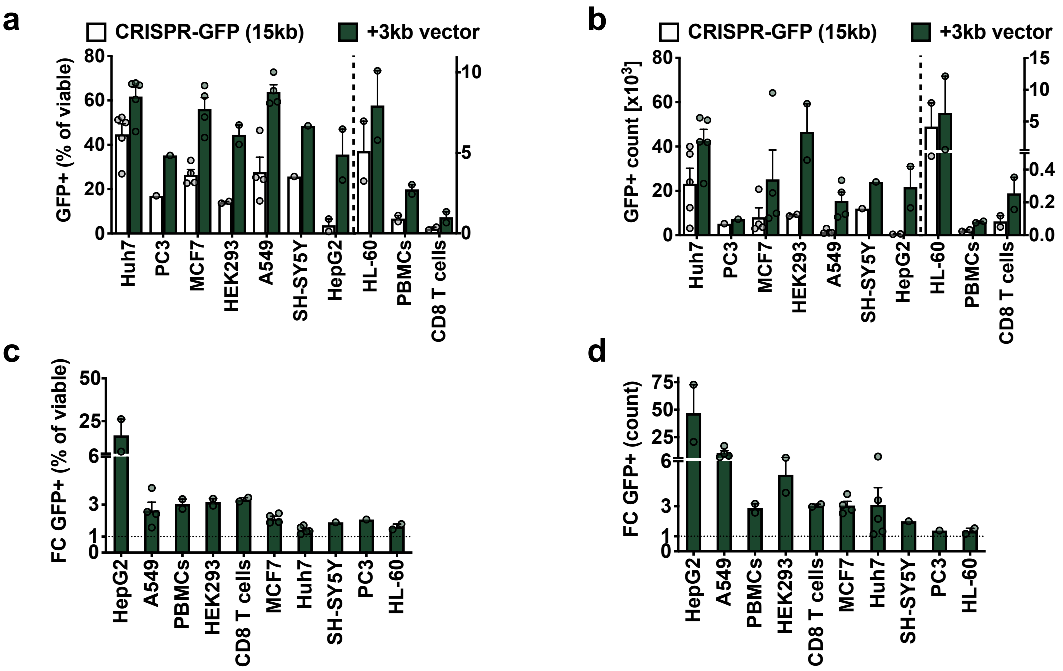

Supplementary Fig. 6: Co-transfection of small vectors increase efficiency in numerous cell types. **a-b**, Bar graphs depicting (**a**) the percent increase of transfection efficiency and (**b**) the total number of GFP+ cells upon co-transfection of the CRISPR-GFP (15kb) vector without (white) or with (dark green) a small vector (3kb) in the tested adherent (left of the dotted line) and non-adherent (right of the dotted line) cells (n=1-6, mean +SEM). Cell types are ordered as in **Fig. 2b**. **c-d**, Bar graphs illustrate the fold change (FC) in the number of (**c**) viable and (**d**) total number of GFP+ cells after adding a small 3kb vector ordered as in **Fig. 2d**.

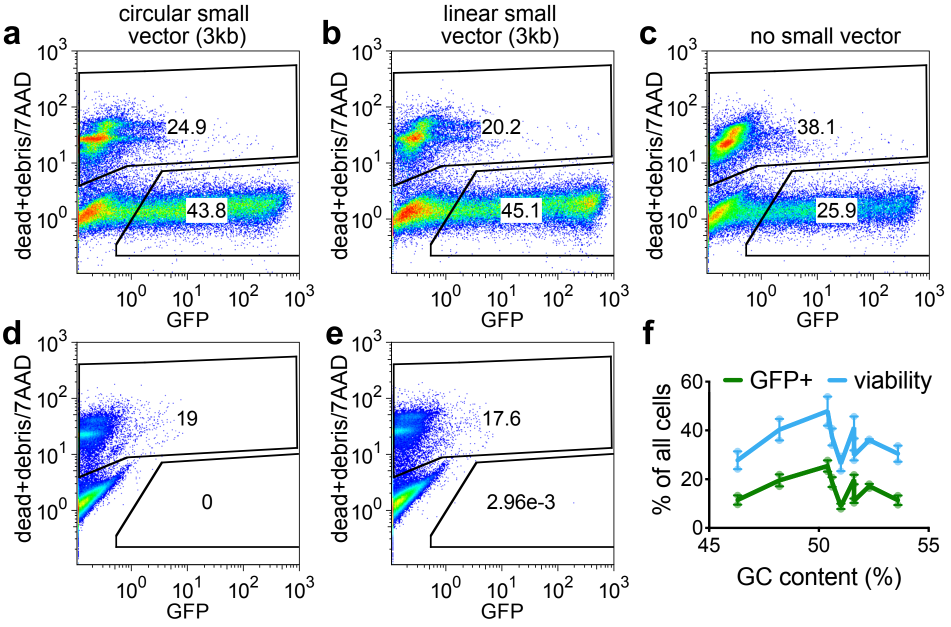

Supplementary Fig. 7: The small vector-mediated increase in transfection efficiency is independent of conformation or sequence content. a-e, Flow cytometry plots show gating of electroporated Huh7 cells. **a-c**, co-transfection of a large CRISPR-GFP vector (15kb) with either (**a**) a circular, (**b**) a linearized (by restriction endonuclease) or (**c**) without a small vector. **d-e**, transfection of either (**d**) a circular or (**e**) a linearized small vector without a large CRISPR-GFP vector. **f**, Line graph illustrates absolute percentage transfection efficiency (green) and cell viability (blue) upon co-transfection of a large CRISPR-GFP vector (15kb) with small vectors of varying GC content in A549 and MCF7 cells (n=2, mean +/- SEM).

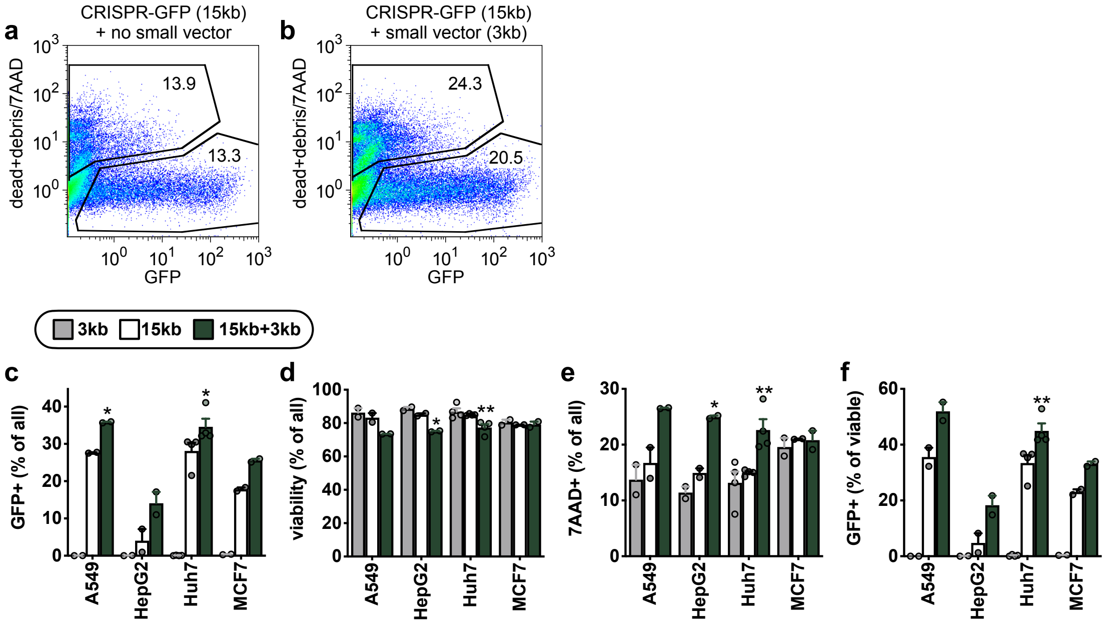

Supplementary Fig. 8: Liposomal transfection efficiency can be improved by co-transfecting large CRISPR vectors with small vectors. a-b, Flow cytometry plots show gating of HepG2 cells 48h after Lipofectamine 3000 transfection of a large CRISPR-GFP vector (15kb) (**a**) without and (**b**) with a small vector (3kb). **c-f**, Bar graphs showing increase in (**c,f**) transfection efficiency (GFP+) and (**d-e**) number of viable and 7AAD+ cells (dead and debris) for Huh7, A549, MCF7, and HepG2 cells upon Lipofectamine 3000 transfection of a CRISPR-GFP vector (15kb) without (white) and with (green) a small vector (3kb). Cell transfection control with only the small 3kb vector is included (grey). Statistics: paired two-tailed t-test, *$p < 0.05$, **$p < 0.01$.
