# Supplementary material for: Successful delivery of large-size CRISPR/Cas9 vectors in hard-to-transfect human cells using small plasmids
Source: Commun Biol. 2020 Jun 19;3:319. doi: 10.1038/s42003-020-1045-7 (PMC7305135; doi:10.1038/s42003-020-1045-7)
Supplement: Supplementary file 2 — Description of Additional Supplementary Files [file 42003_2020_1045_MOESM2_ESM.pdf]

Description of Additional Supplementary Files

File Name: **Supplementary Data 1**
Description: Source data underlying plots shown in figures.
