# Supplementary material for: Successful delivery of large-size CRISPR/Cas9 vectors in hard-to-transfect human cells using small plasmids
Source: Commun Biol. 2020 Jun 19;3:319. doi: 10.1038/s42003-020-1045-7 (PMC7305135; doi:10.1038/s42003-020-1045-7)
Supplement: Supplementary file 5 — Reporting Summary [file 42003_2020_1045_MOESM5_ESM.pdf]

nature research

Corresponding author(s): Claudia Kutter

Last updated by author(s): May 16, 2020

Reporting Summary

Nature Research wishes to improve the reproducibility of the work that we publish. This form provides structure for consistency and transparency in reporting. For further information on Nature Research policies, see Authors & Referees and the Editorial Policy Checklist.

Statistics

For all statistical analyses, confirm that the following items are present in the figure legend, table legend, main text, or Methods section.

n/a	Confirmed	
☐	☒	The exact sample size (*n*) for each experimental group/condition, given as a discrete number and unit of measurement
☐	☒	A statement on whether measurements were taken from distinct samples or whether the same sample was measured repeatedly
☐	☒	The statistical test(s) used AND whether they are one- or two-sided<br>*Only common tests should be described solely by name; describe more complex techniques in the Methods section.*
☒	☐	A description of all covariates tested
☒	☐	A description of any assumptions or corrections, such as tests of normality and adjustment for multiple comparisons
☐	☒	A full description of the statistical parameters including central tendency (e.g. means) or other basic estimates (e.g. regression coefficient) AND variation (e.g. standard deviation) or associated estimates of uncertainty (e.g. confidence intervals)
☒	☐	For null hypothesis testing, the test statistic (e.g. *F*, *t*, *r*) with confidence intervals, effect sizes, degrees of freedom and *P* value noted<br>*Give P values as exact values whenever suitable.*
☒	☐	For Bayesian analysis, information on the choice of priors and Markov chain Monte Carlo settings
☒	☐	For hierarchical and complex designs, identification of the appropriate level for tests and full reporting of outcomes
☒	☐	Estimates of effect sizes (e.g. Cohen's *d*, Pearson's *r*), indicating how they were calculated

Our web collection on statistics for biologists contains articles on many of the points above.

Software and code

Policy information about availability of computer code

Data collection	Transfection efficiency and cell viability was measured by flow cytometry (FACSNavios, Beckman Coulter, Navios Cytometry List Mode Data Acquisition and Analysis Software version 1.3) by gating cells for GFP and 7AAD (FlowJo version 8.2)
Data analysis	The data were plotted and analysed with GraphPad Prism (version 8.4.2), Microsoft Excel (version 16.37), R (version 3.6.1), and ggplot2 (version 3.3.0)

For manuscripts utilizing custom algorithms or software that are central to the research but not yet described in published literature, software must be made available to editors/reviewers. We strongly encourage code deposition in a community repository (e.g. GitHub). See the Nature Research guidelines for submitting code & software for further information.

Data

Policy information about availability of data

All manuscripts must include a data availability statement. This statement should provide the following information, where applicable:
- Accession codes, unique identifiers, or web links for publicly available datasets
- A list of figures that have associated raw data
- A description of any restrictions on data availability

All data generated or analyzed during this study are included in the article. Raw data for graphs can be found in Supplementary Data 1. Additionally, all relevant data are available from the authors upon request.

Field-specific reporting

Please select the one below that is the best fit for your research. If you are not sure, read the appropriate sections before making your selection.

☒ Life sciences ☐ Behavioural & social sciences ☐ Ecological, evolutionary & environmental sciences

For a reference copy of the document with all sections, see nature.com/documents/nr-reporting-summary-flat.pdf

Life sciences study design

All studies must disclose on these points even when the disclosure is negative.

Sample size	Instead of repeatedly testing the same cell line, we tested our approach in 8 different cell lines, and 2 types of primary cells. Most cell lines were assayed at 2-3 different passages.
Data exclusions	No data were excluded from the analysis
Replication	All attempts to replicate were successful. Each new cell line we tested gave the same results!
Randomization	These are cell lines and randomization is not possible.
Blinding	These are cell lines and blinding is not possible.

Reporting for specific materials, systems and methods

We require information from authors about some types of materials, experimental systems and methods used in many studies. Here, indicate whether each material, system or method listed is relevant to your study. If you are not sure if a list item applies to your research, read the appropriate section before selecting a response.

Materials & experimental systems

n/a	Involved in the study	
☒	☐	Antibodies
☐	☒	Eukaryotic cell lines
☒	☐	Palaeontology
☒	☐	Animals and other organisms
☐	☒	Human research participants
☒	☐	Clinical data

Methods

n/a	Involved in the study	
☒	☐	ChIP-seq
☐	☒	Flow cytometry
☒	☐	MRI-based neuroimaging

Eukaryotic cell lines

Policy information about cell lines

Cell line source(s)	ATCC
Authentication	To ensure authenticity, cell lines were initially genotyped by short-tandem repeat genetic profiling (STR) using the PowerPlex_16HS_Cell Line panel and analyzed using Applied Biosystems Gene Mapper ID v3.2.1 software by the external provider Genetica DNA Laboratories (LabCorp Specialty Testing Group) and continuously assessed phenotypically.
Mycoplasma contamination	All cell lines were mycoplasma free when periodically tested with Mycoplasmacheck (Eurofins Genomics) or MycoProbe (R&D)
Commonly misidentified lines (See ICLAC register)	None of the cell lines are listed as misidentified in version 10

Human research participants

Policy information about studies involving human research participants

Population characteristics	Buffy coats were obtained from anonymous healthy volunteers after informed consent and according to institutional guidelines (Karolinska University Hospital, Stockholm, Sweden).
Recruitment	Buffy coats were obtained from anonymous healthy volunteers after informed consent and according to institutional guidelines (Karolinska University Hospital, Stockholm, Sweden).

Ethics oversight | Buffy coats were obtained from anonymous healthy volunteers after informed consent and according to institutional guidelines (Karolinska University Hospital, Stockholm, Sweden).

Note that full information on the approval of the study protocol must also be provided in the manuscript.

Flow Cytometry

Plots

Confirm that:

- [x] The axis labels state the marker and fluorochrome used (e.g. CD4-FITC).
- [x] The axis scales are clearly visible. Include numbers along axes only for bottom left plot of group (a 'group' is an analysis of identical markers).
- [x] All plots are contour plots with outliers or pseudocolor plots.
- [x] A numerical value for number of cells or percentage (with statistics) is provided.

Methodology

Sample preparation	Transfection efficiency was measured after 24h (unless stated differently) by flow cytometry (FACSNavios, Beckman Coulter, Navios Cytometry List Mode Data Acquisition and Analysis Software version 1.3) by gating cells for GFP and 7AAD (FlowJo version 8.2, Supplementary Fig. 1). In order to collect all potentially dead cells, both the supernatants and the adherent cells (harvested by trypsinization) were collected. Cells were washed in 1xPBS with 1% BSA (Sigma), followed by staining in 100 µL buffer with 5 µL 7AAD viability staining solution (eBioscience) for 15 min on ice in the dark. Cells were acquired directly without washing away the staining buffer by flow cytometry.
Instrument	FACSNavios (Beckman Coulter).
Software	FACSNavios, Beckman Coulter, Navios Cytometry List Mode Data Acquisition and Analysis Software version 1.3. The samples were analyzed using FlowJo version 8.2
Cell population abundance	See Fig. 1 and Fig. 2, Supplementary Fig. 1-8
Gating strategy	See Supplementary Fig. 1

- [x] Tick this box to confirm that a figure exemplifying the gating strategy is provided in the Supplementary Information.
